# In-Plane Thermal Diffusivity Measurements of Polyethersulfone Woven Textiles by Infrared Thermography

**DOI:** 10.3390/s22030940

**Published:** 2022-01-26

**Authors:** Mariacristina Larciprete, Noemi Orazi, Yves-Simon Gloy, Stefano Paoloni, Concita Sibilia, Roberto Li Voti

**Affiliations:** 1 Dipartimento di Scienze di Base ed Applicate per l’Ingegneria, Sapienza Università di Roma, Via Antonio Scarpa 16, 00161 Rome, Italy; mariacristina.larciprete@uniroma1.it (M.L.); concita.sibilia@uniroma1.it (C.S.); 2 Dipartimento di Ingegneria Industriale, Università degli Studi di Roma Tor Vergata, Via del Politecnico 1, 00133 Rome, Italy; noemi.orazi@uniroma2.it (N.O.); stefano.paoloni@uniroma2.it (S.P.); 3 Gherzi Germany GmbH, 32780 Chemnitz, Germany; y.gloy@gherzi.de

**Keywords:** lock-in thermography, thermal diffusivity, smart textiles, polyethersulfone, thermal anisotropy

## Abstract

Lock-in thermography was applied to the measurement of the in-plane thermal diffusivity of three polyethersulfone (PES) textiles characterized by different weaving pattern as well as different mass density of interlacing fibers. The experimental results showed that the in-plane thermal diffusivity in each direction decreased with the increase of the fibers’ linear mass density, thus leading to an anisotropic behavior of the thermal diffusivity in the specimen where PES fibers with different density were interlaced. A new theoretical model for the study of the heat diffusion in textiles was specifically developed and, thereafter, employed for the analysis of the experimental results. As such, our textile model approach, shedding light on the role of different textile and fibers parameters on the resulting thermal diffusivity, paves the way for the development and design of textiles with tailored thermal behavior.

## 1. Introduction

Over the recent period, there has been growing interest in knitted fabrics for the realization of electrically driven functional fabrics, so-called smart textiles. Such an interest is due to several technological advances such as the emergence of conductive and elastic yarns for stretchable electronics, the development of conductive inks as well as advances in miniaturization and printing techniques. In this respect, special attention has been devoted to the development of flexible patch antennas and electronics for wearable telemedicine and defense applications [1,2,3], as well as power-assisted garments for personal thermal management. Thanks to their peculiar properties including flexibility and light weight, such devices can be easily integrated into textiles, thus allowing the manufacturing of smart clothes. As regards the production of textile antennas, different fabric materials such as cotton, polyester and nylon are typically employed for the realization of the substrate, while copper adhesive tape, silver conductive ink or conductive paper are usually adopted as radiating materials, to name some [4,5].

Among different material textiles, polyethersulfone (PES) is the most widely employed due to its relevant physical properties. In particular, PES allows the realization of textiles where the fibers are interlaced according to different geometries as those, for instance, required for the realization of integrated antennas. However, several problems remain unsolved and, among others, the dissipation of the heat produced by the integrated electronic devices through the substrate textile is among the most important issue, which still deserves further studies in order to optimize the effective thermal diffusivity and infrared emissivity of these structures [6,7,8].

Fabrics can be viewed as consisting of repeated units of porous yarns and air spaces. Therefore, the heat flow through textiles depends on several factors such as the thermal conductivity of the fibrous material, air volume content and weave pattern, i.e., the way the warp and weft yarns interlace with each other. It is thus evident that one of the fundamental issues to be addressed in the design of smart textiles is to understand how the microscale thermophysical properties of the single bundle of fibers may affect the effective properties of textiles on the macroscopic scale.

Given the considerations reported above, the characterization of the thermal properties of PES textiles is of crucial importance. In this respect, it is worth mentioning that both mechanical, electrical [9] and thermal [10] properties of fabrics have been shown to be extremely anisotropic and, consequently, woven textiles are also expected to exhibit anisotropic thermal transport properties which are significantly affected by fiber structure. In particular, the heat produced by the current flowing into the electrically conductive yarns has been shown to be mainly spread in the plane of the fibers rather than in the perpendicular direction [11,12]. In this regard, it has been recently reported that the presence of anisotropy in the fiber-woven structure [9,13,14], such as those due to different density of warp and weft yarns [15] or yarn count differences in warp and weft directions [16], may lead to a significant anisotropic behavior of the thermal transport properties.

The main idea of this study is to get some further insights about the dependence between the textiles features and the macroscopic heat conduction properties. To this aim, thermal diffusivity measurements have been carried out in PES textiles characterized by different fiber density and weaving pattern. Among different textile thermal properties, thermal diffusivity *D* plays a crucial role in the determination of the transient thermal response of textile material resulting from time-varying heat flow within the material. Thermal diffusivity is a material-specific property defined as *D* = *k*/(*ρC_p_*), where k is the thermal conductivity, *C_p_* is the isobaric specific heat and *ρ* the mass density, which basically determines the rate of heat transfer in a medium.

In this study, the thermal diffusivity measurements have been carried out by means of active infrared thermography (IRT) technique, which nowadays can be considered one of the most well-established techniques for the remote and nondestructive evaluation of materials thermal properties. In particular, in these investigations the lock-in IRT configuration (LI-IRT) [17] has been employed since it allows the straightforward evaluation of the in-plane thermal diffusivity. As mentioned before, such an information can be extremely useful when taking into account that heat dissipation in smart textiles is expected to take place mainly along the sample surface rather than in the orthogonal direction. In addition, the LI-IRT technique enables the prompt evaluation of the thermal diffusivity along different directions on the sample surface [18,19] and hence, the detection of a possible anisotropic features of the thermal diffusivity, which may result from the textile physical properties and geometrical structure, which can be optimized and exploited for smart design processes.

## 2. Materials and Methods

### 2.1. Materials

**Figure 1:**
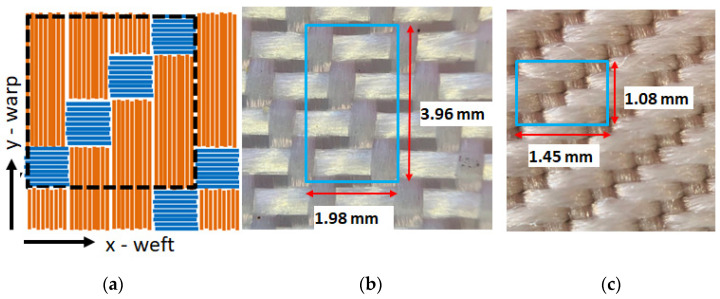
Sketch of the 3:1 twill weaving pattern (**a**); optical microscopy image of PES1 (**b**) and PES2 (**c**) samples, where the size of the unit cell adopted in the simulations has been highlighted.

**Figure 2:**
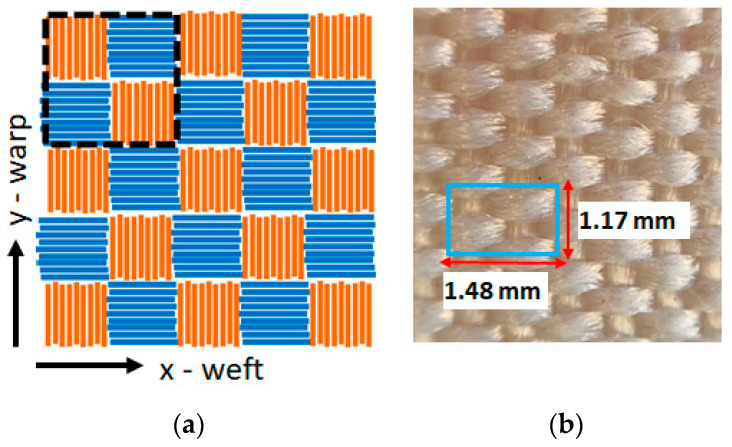
Sketch of the 1:1 plain weaving pattern (**a**); optical microscopy image of PES3 sample (**b**). The size of the unit cell used in the simulations is highlighted in the blue box.

The typical woven texture is characterized by an orthogonal interlacement of the weft and warp yarns, which are oriented in a parallel (*x*-axis) and orthogonal (*y*-axis) direction to the textile main length, respectively. In a plain weave, the warp and weft are interlaced according to a crisscross pattern where the weft yarns alternately pass over and under the warp yarns. As shown by the sketches in Figure 1 and Figure 2, the investigated PES samples are characterized by two different weaving patterns, i.e., 3:1 (twill weave: see Figure 1a) and 1:1 (plain weave: see Figure 2a), corresponding to the number of warp threads versus weft threads.

Two sets of PES fibers characterized by different linear mass density, i.e., 334 dtex and 1100 dtex, respectively, have been employed for the realization of the investigated samples. The optical microscopy images of the investigated samples are shown in Figure 1 and Figure 2, where the woven structure consisting of repeated basic units with a lateral size of the order of a few millimeters can be clearly identified. For the sake of clarity, the properties of the investigated samples have been summarized in Table 1.

### 2.2. LI-IRT Technique

IRT is a remote and nondestructive technique that is employed in a large variety of different fields such as material properties evaluation [20] or cultural heritage investigations [21,22]. IRT relies on detecting the transient temperature time-variation that is produced in the investigated sample by the absorption of either pulsed or periodically intensity-modulated visible light beam. The induced temperature distribution depends not only on the amount of absorbed light intensity, but also on the sample’s geometrical and thermal properties. Therefore, the detection of such a temperature variation can constitute a valuable tool to gather information on the above-mentioned sample properties. In the IRT technique, the detection is carried out by means of an infrared camera that provides a sequence of images referred to as thermograms.

As regards the thermal diffusivity evaluation, IRT can be applied in different experimental configurations [17]. In the LI-IRT technique both the sample heating and the detection of the induced temperature distribution take place at the same sample surface. More specifically, an intensity-modulated focused laser beam is adopted to locally heat the sample and, hence, to generate an oscillating temperature distribution. A synchronized infrared camera provides the phase images of the oscillating temperature variation at the sample surface which are especially suited for the determination of the thermal diffusivity. In fact, in thermally thick opaque samples the phase *ϕ(r)* shows an asymptotic linear dependence on the lateral distance *r* from the heating beam incident point which can be expressed as:

(1) $$\phi (r)=\phi_{0}-\frac{r}{\mu }$$

where *ϕ_0_* is a constant while *μ* = $\sqrt{\left(D/\pi F\right)}$ is the thermal diffusion length. Here *f* is the heating beam modulation frequency. Therefore, the thermal diffusivity can be readily estimated from the slope Δ = *dϕ*/*dr* as follows [23,24,25,26]:

(2) $$D=\pi f/\Delta^{2}$$

**Figure 3:**
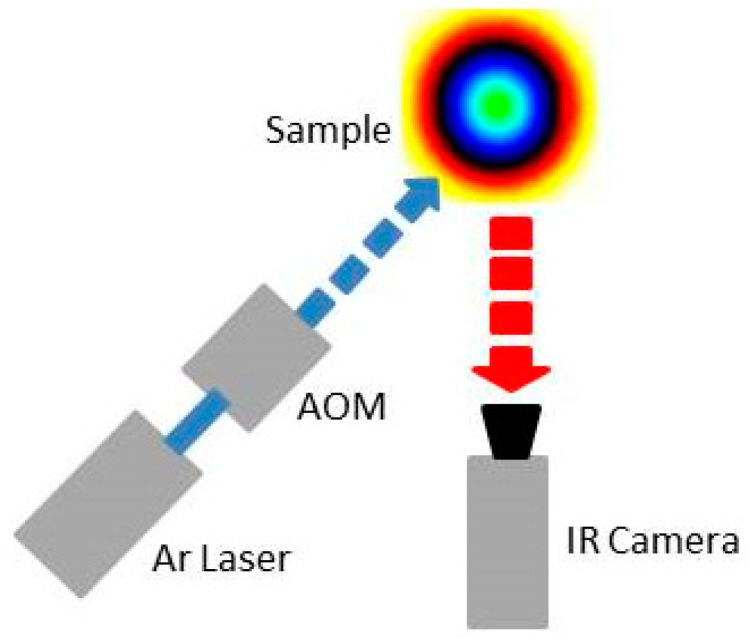
Sketch of the LI-IRT experimental set up.

As depicted in Figure 3, in the adopted experimental set up, an Ar laser beam focused on a spot of the order of 30 μm was employed as a 150 mW heating beam. The beam intensity was square-wave-modulated at *f* = 0.02 Hz by means of an acousto-optic laser beam modulator (AOM). A signal generator was used to both drive the AOM and to provide the reference signal for the lock-in acquisition system. The detection of the temperature distribution at the heated sample surface was carried out by means of an infrared camera (CEDIP Jade MWIR) operating in the mid-infrared wavelength range (3.5–5.1 µm) with an acquisition frame rate of 150 Hz. The infrared detection system of such a camera is based on a 320 × 240 pixel InSb focal plane array, with a pixel pitch of 30 μm and a thermal sensitivity corresponding to a noise-equivalent temperature difference (NETD) of 25 mK at 300 K.

## 3. Results and Discussion

### 3.1. Experimental Results

**Figure 4:**
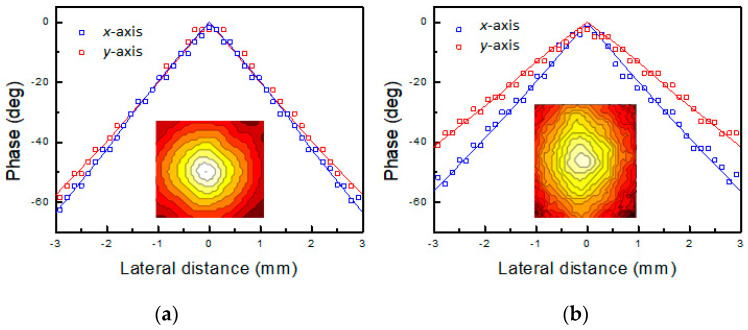
Signal phase profiles obtained along the *x*-axis (blue curve) and *y*-axis (red curve) in PES1 (**a**) and PES3 (**b**) samples. In the inset, the corresponding LI-IRT phase images are shown.

The IRT-LI phase signal vs. lateral distance for PES1 and PES3 samples are reported in Figure 4a,b, respectively, where the insets display the corresponding phase contour maps. In sample PES2, phase contour maps (not shown) similar to the PES1 ones were also obtained.

The phase images corresponding to the samples having the same yarn density on both warp and weft direction, i.e., PES1, consist of nearly circular contour lines (see the inset in Figure 4a). Such a result is consistent with an isotropic behavior of the thermal diffusivity. In fact, in thermally isotropic media, the localized laser beam heating gives rise to spherical thermal waves, which diffuse through the sample volume. Consequently, the surface temperature isophases consist of concentric and equidistant circumferences.

The corresponding phase vs. lateral offset plots along the *x*- and *y*-axis are shown in Figure 4a where the isotropic character of the thermal diffusivity can be clearly observed from the almost identical slope of the two data sets.

Unlike the PES1 one, the phase image obtained in the PES3 sample consists of concentric and equidistant ellipses (see the inset in Figure 4b), thus clearly indicating a corresponding anisotropic character of the thermal diffusivity. Here, the thermal diffusivity values for different directions along the sample surface can be evaluated by means of the corresponding values of the linear slope of the phase *ϕ*(*r*) with the lateral offset *r*. In Figure 4b the phase profiles obtained along the *x*- and *y*-axis are reported, where the anisotropic behavior of the thermal diffusivity is evidenced by the different slopes of the two profiles.

The thermal diffusivity values for the investigated samples retrieved along both the *x*- and *y*-axis by means of Equation (2) are summarized in Table 2. In particular, the results obtained in samples PES1 and PES2 show a similar value of *D* along the *x* and *y* directions despite their asymmetric weaving pattern (3:1). On the other hand, the diffusivity values are significantly affected by the fibers’ linear mass density. A larger thermal diffusivity value was obtained in the sample made of lower density fibers.

As a preliminary remark, the obtained thermal diffusivity values are larger than the ones reported in the literature [20,27,28,29] for bulk PES (*D* ~ 0.1 mm^2^/s). One reason is due to the effective mass density of the investigated PES fibers that can be expected to be lower than the bulk PES one, thus leading to a larger value for the thermal diffusivity. In addition, in sample PES3 the thermal diffusivity shows a remarkable anisotropic behavior due to the different fiber density in the warp and weft yarns. Similar to PES1 and PES2, in PES3 the thermal diffusivity increases with decreasing fiber density values.

As regards previous in-plane thermal diffusivity studies carried out by means of IR-LIT, an anisotropic thermal diffusivity distribution was also found in woven carbon reinforced polymers (CFRP), where the measured *D* values were larger along the carbon fiver direction [14,19]. Similar results were obtained in a plastically deformed steel specimen where the anisotropy was due to the alignment of the grains along the loaded direction [20].

### 3.2. Theoretical Model

In order to analyze the experimental results, a specifically designed mathematical model to study the heat diffusion taking place in textiles was introduced. The thermal diffusivity obtained from simulations was eventually compared with the experimental results in order to achieve a better understanding of the interplay among the fibers’ properties, textiles weaving pattern and the resulting macroscopic thermal properties.

In this study a finite difference time domain (FDTD) model for the solution of the Fourier heat diffusion equation was developed [30,31]. Such a model was then used to derive the ac temperature distribution produced by the absorption of a modulated laser beam in textiles. Since the typical thickness value of textiles is less than a few millimeters, in this model the induced temperature variation was assumed to depend only on the coordinates parallel to the sample surface, i.e., *x* and *y*. Following this approach, textile structures were considered as made of the periodic dispositions of a basic unit cell, each one being made of 2D pixels corresponding to weft or warp threads portions where the thermal properties can be considered homogeneous. The values of the lateral size of such 2D pixel reported in Table 1 were estimated on the basis of the highlighted sample areas in Figure 1 and Figure 2.

**Figure 5:**
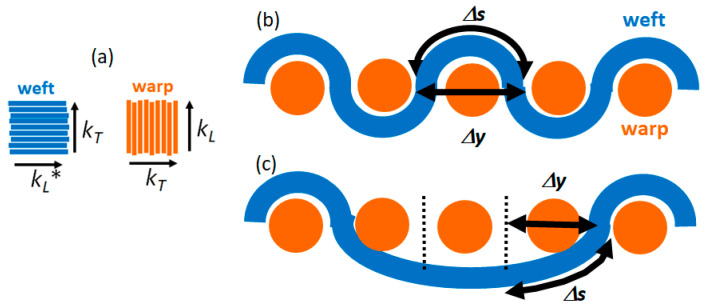
Schematic representations showing the transversal and longitudinal thermal conductivity in weft and warp yarns (**a**); cross section of the 1:1 (**b**) and 3:1 (**c**) weaving patterns.

Each 2D pixel is characterized by an anisotropic thermal conductivity whose value depends on whether the pixel belongs to a weft or a warp thread (see Figure 5a). As regards the warp threads, the longitudinal thermal conductivity value along the bundles’ direction *k_L_* is likely to be in the range of values reported in the literature for PES fibers (0.13 ÷ 0.18 W/mK [28,29]), while for the transversal component corresponding to the orthogonal direction to the PES fibers, i.e., *k_T_*, a slightly lower value was assumed, since the heat is expected to flow more effectively along the fibers than across them. The same considerations cannot be applied to the longitudinal value of the thermal conductivity of the weft yarns. Unlike the warp ones, the weft yarns are arranged along a curved path passing over and under the warp yarns (see Figure 5b,c). Therefore, if one assumes the yarn’s thermal resistance is proportional to its length, the equivalent thermal conductivity value can be expressed as k_L_* = *δ∙k_L_*, where *δ* = Δ*y*⁄Δ*s* depends on the specific weaving pattern as shown in Figure 5b,c. Based on geometrical considerations, *δ* was estimated to be equal to 0.3 and in the 0.3 < *δ* < 0.9 range for the 1:1 and 3:1 weaving patterns, respectively. Finally, the value of the transversal component of the thermal conductivity *k_T_* value was considered equal to the corresponding value for the warp yarns.

Bearing in mind the assumptions reported above, the discretized time domain Fourier heat diffusion equation can be written as

(3) $$T_{i,j}\left(t+\Delta t\right)=T_{i,j}\left(t\right)+\left[kx_{i,j}\frac{T_{i+1,j}\left(t\right)-2T_{i,j}\left(t\right)+T_{i-1,j}\left(t\right)}{\Delta x^{2}}+ky_{i,j}\frac{T_{i,j+1}\left(t\right)-2T_{i,j}\left(t\right)+T_{i,j-1}\left(t\right)}{\Delta y^{2}}+w_{i,j}\left(t\right)\right]\frac{\Delta t}{\rho_{i,j}C_{p}}$$

where *i*,*j* are the indexes of a generic 2D pixel located at the position (*x_i_* = *i*Δ*x*; *y_i_* = *j*Δ*y*), *T*_*i*,*j*_(*t*) is the corresponding temperature time variation, *kx*_*i*,*j*_ and *ky*_*i*,*j*_ represent the pixel thermal conductivity values along the *x*- and *y*-axis that were still considered parallel to the weft and warp direction, respectively, *ρ*_*i*,*j*_ is the pixel mass density, *C_p_* is the isobaric specific heat and *w*_*i*,*j*_ is the heat power density, which was considered different from zero only at the center of the grid. Such an equation was integrated over a long enough time interval in order to make the transient response negligible, following the application of the heating laser beam at *t* = *0* and, hence, to obtain the steady-state component of *T*_*i*,*j*_(*t*). In these simulations, the isobaric specific heat for polyethersulfone was set to the literature values *C_p_* = 1.1 kJ kg^−1^ K^−1^ [28,29]. As mentioned above, the mass density of the fiber bundles is expected to be lower than the PES bulk value *ρ_o_* = 1370 kg/m^3^ [28,29] due to the empty spaces between the fibers. Owing to this reason, in Equation (3) an effective density value *ρ*_*eff*_ = *f*∗*ρ_ο_* was assumed, where *f* corresponds to the volume filling factor of the fibers inside the bundle. As discussed in the next paragraph, from the analysis of the experimental data, a filling factor *f* = 20% and *f* = 9% were evaluated in the PES1 and PES2 samples, respectively. Consequently, the numerical simulations were carried out by alternating for each 2D pixel the local value of the filling factor (*f* = 9% or 20%) corresponding to the type of yarn (see Figure 2a).

**Figure 6:**
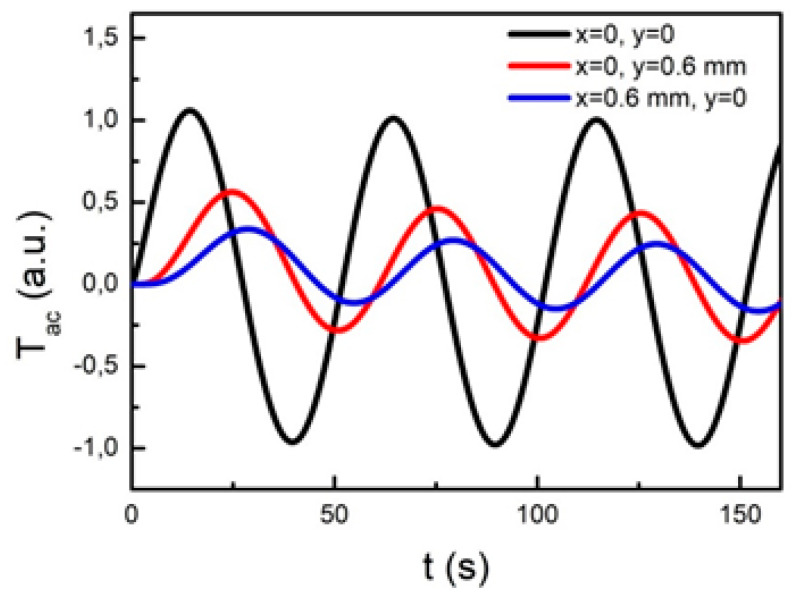
Time dependence of the ac temperature variation obtained in the PES3 sample for the different positions reported in the inset.

As an example, Figure 6 shows the temperature time dependence induced by the absorption of a modulated laser beam at *f* = 0.02 Hz for three different 2D pixels belonging to the same textile where the 1:1 weaving pattern was assumed. One pixel corresponds to the laser irradiated spot at the center of the grid (black curve) while the other two are placed 0.6 mm away from the first one along the *x*-axis (blue curve) and the *y*-axis (red curve), respectively. In these simulations, the thermal diffusivity values along the *x-* and *y*-axis were considered equal to the ones obtained for sample PES3 (see Table 1).

As expected, after a long enough time following the onset of the heating beam such that the transient response can be neglected, all curves harmonically oscillate at the same period (*T* = 50 s) of the heating beam. The time shift of the red and blue curves in comparison to the black one is due to the time needed by the induced heat to diffuse from the irradiated sample spot up to the considered pixels. Even if the two pixels are located at the same distance from the heating beam, the red curve is shifted to a larger extend in comparison with the blue one because of the lower value of the thermal diffusivity along the *x*-axis (see Table 1) in comparison to the one along the *y*-axis, thus highlighting the anisotropic feature of the thermal transport properties. Owing to the same reason, the red curve shows a larger amplitude than the blue one because of the attenuation undergone by the induced temperature distribution while diffusing through the sample, the attenuation being more severe for decreasing values of the thermal diffusivity. As shown later on, from the time delay values obtained for pixels located at varying distances *r* from the heated spot along a given direction, the phase shift *ϕ*(*r*) of the induced temperature oscillation and, consequently, the corresponding value of the thermal diffusivity can be readily evaluated.

### 3.3. Data Analysis and Discussion

As shown in the previous paragraph, in samples PES1 and PES2, an isotropic behavior of the thermal diffusivity was unexpectedly observed despite the asymmetric weaving pattern of the two textiles. In order to account for such a circumstance, the effective thermal conductivity of the 2D pixels constituting the 3:1 pattern was theoretically evaluated along both the *x*-axis (weft) and *y*-axis (warp). In these simulations, 3:1 textile structures were considered made of the periodic dispositions of a basic unit cell, each made of 2D pixels as highlighted by the dashed box in Figure 1 and Figure 2, where the possible thermal contact resistance among adjacent 2D pixels was neglected. The effective thermal conductivity along the *x*(*y*)-axis can be considered as the series of the thermal conductivity along the *x*(*y*) direction of the unitary cell and, therefore, it can be obtained as follows:

(4) $$\{\begin{array}{c} \frac{1}{k_{x}}=\frac{1}{m+1}\sum_{i}^{m+1}\frac{1}{k_{x,i}}=\frac{1}{m+1}\left(\frac{1}{\delta \cdot k_{L,}}+\frac{m}{k_{T}}\right)\\ \frac{1}{k_{y}}=\frac{1}{m+1}\sum_{i}^{m+1}\frac{1}{k_{y,i}}=\frac{1}{m+1}\left(\frac{1}{k_{T}}+\frac{m}{k_{L}}\right) \end{array}$$

leading to

(5) $$\{\begin{array}{c} k_{x}=\frac{\left(m+1\right)\delta \cdot k_{L}\cdot k_{T}}{k_{T}+m\cdot \delta \cdot k_{L}}\\ k_{y}=\frac{\left(m+1\right)k_{L}\cdot k_{T}}{k_{L}+m\cdot k_{T}} \end{array}$$

where the weaving texture pattern is generically assumed to be *m*:1.

From Equation (5) the ratio of the thermal diffusivity values along the x- and y-axis, respectively, can be obtained as follows

(6) $$\frac{D_{y}}{D_{x}}=\frac{k_{y}}{k_{x}}=\frac{k_{T}+m\cdot \delta \cdot k_{L}}{\delta \left(k_{L}+mk_{T}\right)}$$

from which one may derive the thermal conductivity ratio *k_L_/k_T_* as follows:

(7) $$\frac{k_{L}}{k_{T}}=\frac{m\delta \frac{D_{y}}{D_{x}}-1}{\delta \left(m-\frac{D_{y}}{D_{x}}\right)}$$

In both PES1 and PES2 samples, *m* = 3 and if the amplitude of the weft yarns undulation over the warp ones is considered relatively small such that we can assume *δ* = Δ*x*/Δ*s* = 0.85, a thermal conductivity ratio *k_L_*/*k_T_* ≈ 1.06 can be obtained from Equation (7) despite the almost isotropic thermal diffusivity ratio *D_y_*/*D_x_* ≈ 1.

**Figure 7:**
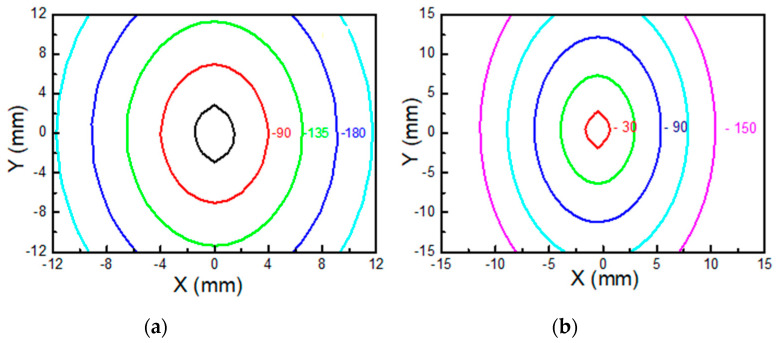
Phase contour plots obtained by means of Equation (3) for the values reported in Table 2 in PES1 (**a**) and PES3 (**b**) samples.

As a confirmation, Figure 7a shows the simulated phase contour plots for sample PES1 obtained by means of Equation (3) for the thermal conductivity values reported in Table 2. The isotropic characteristic of the thermal diffusivity is highlighted by the almost circular shape of the isophase curves that leads to similar slope in the phase vs. lateral displacement profiles along the *x*-axis and *y*-axis.

By means of Equation (5), the following expression for the longitudinal component of the thermal conductivity *k_L_* can be readily obtained

(8) $$k_{L}=\left(\frac{m+\frac{k_{L}}{k_{T}}}{m+1}\right)k_{y}=\left(\frac{m+\frac{k_{L}}{k_{T}}}{m+1}\right)D_{y}C_{p}\left(f\rho_{0}\right)$$

that, for both PES1 and PES2, provides the same value for *k_L_* = 0.16 W/(mK) and, consequently, for *k_T_* = 0.15 W/(mK) (see Table 2), such values being in good agreement with the ones reported in the literature for the polyethersulfone bulk material [28,29].

Concerning sample PES3, the thermal diffusivity anisotropy is likely to be associated with the different dtex density of the weft and warp yarns, respectively. As a preliminary confirmation, if one assumes for the 1100 and the 334 dtex fibers, an effective mass density *ρ_eff_* = *fρ_ο_*_,_ where *f* is equal to 20% and 9%, respectively (see Table 2), a good agreement with the experimental results is obtained by using for PES3 the same *k_L_* and *k_T_* values found in PES1 and PES2, but with a different value of *δ* that can be evaluated by means of Equation (6) as follows:

(9) $$\delta =\frac{1}{m\frac{D_{y}}{D_{x}}-\frac{k_{L}}{k_{T}}\left(m-\frac{D_{y}}{D_{x}}\right)}=0.36$$

In this respect it is worth noting that such a *δ* value corresponds to a relevant out-of-plane undulation of the weft yarns that is consistent with the 1:1 weaving pattern.

In particular, the theoretical predictions obtained by using FDTD (see Equation (3)) give rise to elliptical isophase curves for PES3 as shown in Figure 7b, whose linear dependence on the radial distance along the *x*- and *y*-axis is in agreement with the measured values of the thermal diffusivity.

## 4. Conclusions

In conclusion, lock-in thermography was applied to evaluate the in-plane thermal diffusivity of a set of PES woven textiles along the warp and the weft yarns direction, respectively, to detect the possible presence of anisotropy in the thermal diffusion properties. The obtained thermal diffusivity values were found to be mainly dependent on the fiber density, while, on the contrary, the weaving pattern of the woven structure did not play a crucial role. In fact, in the case of samples characterized by an asymmetric 3:1 weaving pattern, the thermal diffusivity showed an isotropic characteristic because of the identical linear mass density of the warp and weft PES fibers. In order to account for such a circumstance, a theoretical model for the study of the heat diffusion in textiles was developed, where textiles are seen as electrical circuits made of thermal resistors. Such a model enabled us to verify the consistency of an isotropic thermal diffusivity behavior in the case of 3:1 weaving patterns. Unlike the previous one, in the sample characterized by a 1:1 weaving pattern, the thermal diffusivity showed an anisotropic behavior. In particular, the thermal diffusivity measured along the lower mass density warp yarns was larger in comparison with that corresponding to a higher mass density.

More generally, it was shown how lock-in thermography allows the detection of the thermal anisotropy in textiles at a mesoscopic scale [32], and a new theoretical approach was introduced to predict the conditions required to observe in-plane thermal anisotropy. The resulting theoretical model is a useful tool for designing textiles with tailored thermal properties, while machine-learning-based algorithms (neural networks [33], genetic algorithms [34,35] and other optimization search methods [36,37,38,39,40]) can be introduced to further optimize the thermal performance of the designed textile. The results open new ways to design and optimize smart textiles and metasurfaces with controlled anisotropic thermal properties.

The results reported in this work can be considered as the starting point for future improvements concerning the reliability of both the experimental results and the theoretical model. To this aim, further measurements carried out on other kinds of textiles are currently underway, whose results will eventually be compared with the predictions obtained from the refined versions of the theoretical model.

## Figures and Tables

**Table 1:** Main structural and geometrical properties of the investigated PES samples.

Sample	Warp Density (dtex)	Weft Density (dtex)	Weaving Pattern	2D Pixel Δ*x* (mm)	2D Pixel Δ*y* (mm)
PES1	1100	1100	3:1	0.5	1.0
PES2	334	334	3:1	0.36	0.27
PES3	334	1100	1:1	0.74	0.58

**Table 2:** Overview of the results obtained for the investigated PES samples.

Sample	*D_x_* mm^2^/s	*D_y_* mm^2^/s	*D_y_/D_x_*	*f*	*m*	*δ*	*k_L_* Wm^−1^ K^−1^	*k_T_* Wm^−1^ K^−1^	*k_L_/k_T_*
PES1	0.48	0.51	1.063	0.20	3	0.85	0.16	0.15	1.05
PES2	1.07	1.15	1.075	0.09	3	0.85	0.16	0.15	1.06
PES3	0.64	1.20	1.875	0.15	1	0.36	0.16	0.15	1.06

## Data Availability

Research data are not shared.
